# Factors associated with antibiotic initiation and bacterial coinfection in adults with confirmed influenza

**DOI:** 10.1186/s12879-026-13166-0

**Published:** 2026-03-25

**Authors:** Niklas Steger, Erik Isaksson, Anna M. Nordenskjöld, Simon Athlin

**Affiliations:** 1 Department of Infectious Diseases, Faculty of Medicine and Health, Örebro University, Örebro, Sweden; 2 Department of Infectious Diseases, Örebro University Hospital, Örebro, Sweden; 3 Department of Cardiology, Faculty of Medicine and Health, Örebro University, Örebro, Sweden

**Keywords:** Influenza, Emergency department, Antibiotics, Bacterial coinfection

## Abstract

**Background:**

Influenza causes seasonal epidemics worldwide. Bacterial coinfection is a serious complication that, due to diagnostic uncertainty, often results in empirical antibiotic overuse. We aimed to identify clinical and biochemical factors associated with antibiotic initiation and bacterial coinfection in adults with confirmed influenza at the emergency department.

**Methods:**

Adult patients presenting to the emergency department with laboratory-confirmed influenza were included across three influenza seasons (2017–2020). Clinical and biochemical data were collected through medical chart review.

**Results:**

A total of 174 influenza-positive patients were included. Independent predictors of antibiotic initiation were clinical scores NEWS2 ≥ 7 (aOR 8.99, 95% CI 2.38–33.96) and CRB-65 ≥ 2 (aOR 13.22, 95% CI 2.70–64.73), as well as biomarkers CRP (aOR 1.57, 95% CI 1.21–2.02), and white blood cell count (aOR 1.25, 95% CI 1.07–1.46). Only CRP was independently associated with bacterial coinfection (aOR 1.47, 95% CI 1.16–1.86). A CRP threshold ≥ 20 mg/L demonstrated 100% sensitivity for detecting bacterial coinfection.

**Conclusion:**

NEWS2, CRB-65, CRP, and white blood cell count were independently associated with antibiotic initiation in adults with influenza, while only CRP predicted bacterial coinfection. Our findings could improve antibiotic stewardship strategies by underscoring the limited predictive value of clinical scores and the optimal use of biomarkers.

**Clinical trial number:**

Not applicable.

**Supplementary Information:**

The online version contains supplementary material available at 10.1186/s12879-026-13166-0.

## Introduction

Influenza is a major cause of respiratory illness worldwide, contributing substantially to annual morbidity and mortality [1]. Seasonal influenza is estimated to cause 3–5 million hospitalizations and 290,000–650,000 respiratory deaths annually [2, 3]. A considerable proportion of disease burden arises from complications such as bacterial coinfection, which can worsen clinical outcomes and may account for up to 25% of influenza-related deaths [4, 5].

Reverse transcription polymerase chain reaction (RT-PCR) enables accurate diagnosis of influenza within hours [6], but the risk of bacterial coinfection challenges the management of influenza disease as a pure viral infection. Bacterial cultures typically require several days to complete and radiologic imaging is sometimes unavailable due to limited resources or time constraints. Instead, clinicians often rely on clinical signs or biomarkers to assess the risk of bacterial coinfection [7, 8]. Although biomarkers appears superior in differentiating viral from bacterial respiratory tract infection (RTI), no single diagnostic method has demonstrated sufficient reliability [8].

Diagnostic uncertainty likely contributes to antibiotic overprescription in viral RTIs, particularly in emergency departments (EDs) [9]. While RT-PCR-based viral testing has been associated with reduced antibiotic use, a subset of patients with confirmed influenza still receives antibiotics due to concern about bacterial coinfection [10–12]. Given the role of antibiotic overuse in driving antimicrobial resistance, implementing accurate diagnostic methods and strengthening antibiotic stewardship efforts is essential [13].

Few studies have examined factors associated with antibiotic initiation in adult patients at the ED, and none have focused exclusively on those with laboratory-confirmed influenza [11, 12, 14]. As biomarkers appear to predict bacterial coinfection more accurately than clinical signs [8], it is of interest to determine which factors are associated with the antibiotic initiation. Furthermore, research on factors associated with confirmed bacterial coinfection in this population is limited [7, 15, 16]. This prospective study aims to identify clinical and biochemical factors associated with antibiotic use and bacterial coinfection among adults with confirmed influenza in the ED.

## Materials and methods

### Study design and population

This is a post hoc analysis of data collected in a prospective, observational cohort study conducted at the EDs of Örebro University Hospital and Karlskoga Hospital, Sweden. The primary aim of the original study was to investigate cardiac complications in patients with influenza [17]. Patients were recruited over three consecutive influenza seasons from 2017 to 2020. The influenza season was defined annually as October 1 to April 30 [18]. The recruitment was interrupted on 17 February 2020 due to the emerging COVID-19 pandemic.

**Fig. 1:**
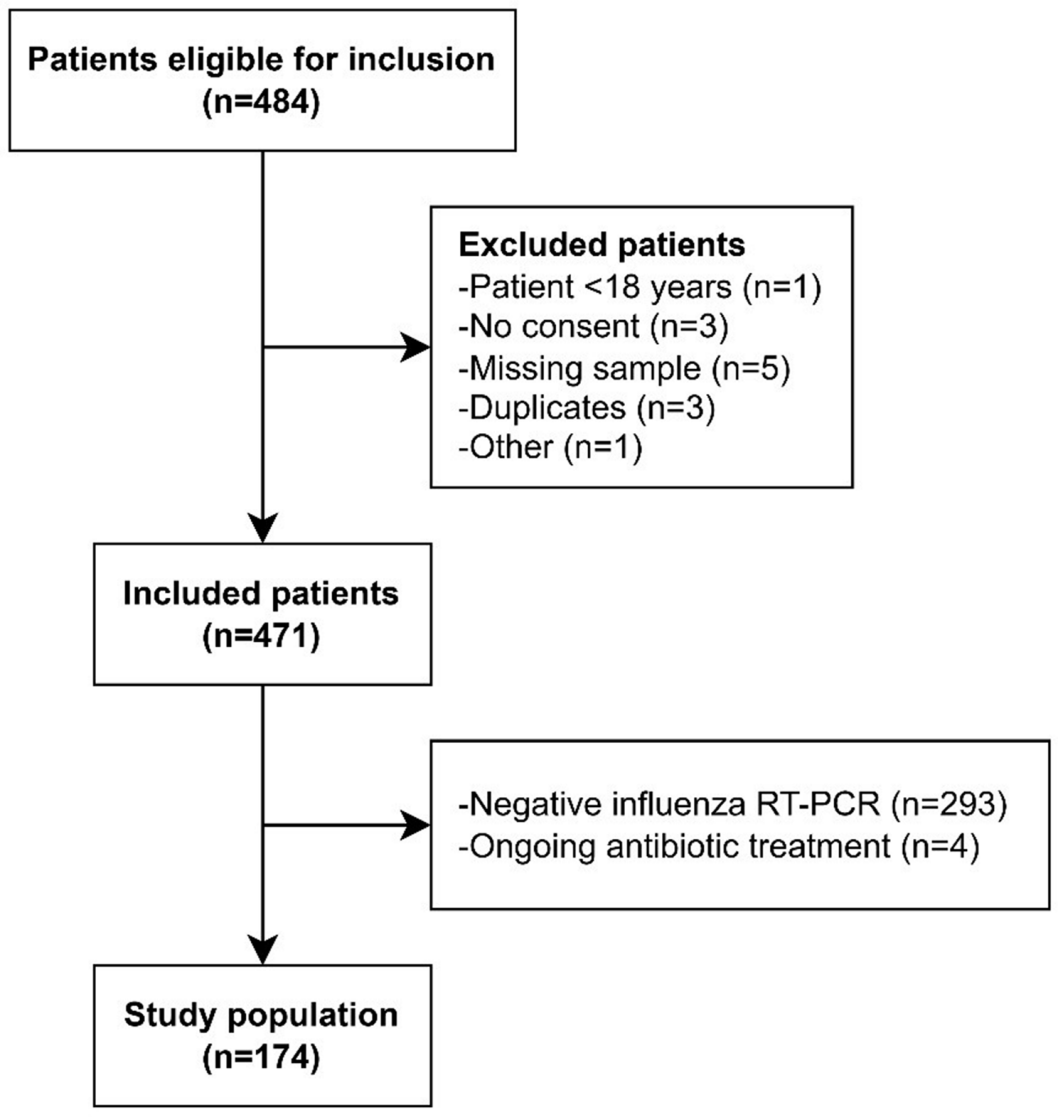
Flowchart describing the enrollment process. RT-PCR: reverse transcription polymerase chain reaction

All patients ≥ 18 years presenting to the ED with clinical suspicion of ongoing influenza and tested with RT-PCR were eligible for inclusion (Fig. 1). Written informed consent was required. Exclusion criteria included inability to provide informed consent or presenting symptoms indicative of acute coronary syndrome, acute heart failure, rapid atrial fibrillation or acute stroke. Of the prospectively included patients, those who had a negative influenza RT‑PCR result and had ongoing antibiotic treatment at the time of inclusion were later excluded. For the bacterial coinfection analysis, only patients with at least one relevant microbiological test were included.

### Data collection

Data on medical history, vaccination status, duration of illness, and clinical symptoms were collected through questionnaires originally developed for the initial cohort study (Additional file 1). Symptom duration < 10 days was considered as associated with influenza based on the World Health Organization surveillance case definition for influenza-like illness [19]. Nasopharyngeal swabs were collected for RT-PCR analysis of influenza and respiratory syncytial virus (Simplexa Flu A/B & RSV, Focus Diagnostics, Cypress, CA, USA). Other microbiological, radiological and laboratory tests were performed according to routine practice in Region Örebro County, as was any treatment provided to the patients.

Additional data were obtained through retrospective medical chart review. Vital signs at ED presentation were used to calculate the National Early Warning Score 2 (NEWS2) and the clinical score for pneumonia based on confusion, respiratory rate ≥ 30, blood pressure systolic < 90 or diastolic ≤ 60 and age ≥ 65 years (CRB-65). Severe illness was defined as NEWS2 ≥ 7 or CRB-65 score ≥ 2 [20, 21]. The first C-reactive protein (CRP) level and white blood cell (WBC) count measured after inclusion were recorded. Data on antibiotic and antiviral initiation at the ED, as well as within seven days before and after inclusion, were recorded. Radiological findings from chest X-ray (CXR) or computed tomography performed within two days of inclusion were recorded.

Microbiological results from blood-, nasopharyngeal and sputum cultures, urinary antigen tests for *Streptococcus pneumoniae* and *Legionella pneumophila*, and RT-PCR panels for atypical respiratory bacteria *(Mycoplasma pneumoniae*,* Chlamydia pneumoniae* and *Chlamydia psittaci)* obtained within seven days before or after inclusion were recorded.

Comorbidities were assessed using a modified Charlson Comorbidity Index (mCCI) [22]. Since data on prior cancer diagnosis, cirrhosis, ongoing dialysis, and acquired immunodeficiency syndrome were not recorded at inclusion, these were completed retrospectively through chart review.

### Endpoint definitions

Primary endpoint was receiving antibiotic treatment at the ED. Antibiotics initiated for indications other than respiratory tract infections were excluded. Secondary endpoint was having a bacterial coinfection, defined as at least one relevant microbiological pathogen detected from any microbiological test performed within 48 h of inclusion. All bacterial findings in blood- or sputum culture, urine antigen tests and PCR tests for atypical bacteria were considered relevant, except for coagulase-negative staphylococci isolated from blood cultures, which were considered contaminants. For nasopharyngeal cultures, only isolation of *S. pneumoniae* or *Haemophilus influenzae* was considered relevant.

### Statistical analysis

Categorical variables were compared using Pearson’s chi-square or Fisher’s exact tests, as appropriate. Continuous variables were compared using the Mann-Whitney U test. For each score, cases with missing data were excluded from the analysis. Statistical significance was set at a two-sided p-value < 0.05. 95% confidence intervals (CIs) were reported. Variables with a univariate p-value < 0.05, along with age and mCCI, were included in a multivariable regression model. Receiver operating characteristic (ROC) curves and corresponding areas under the curve (AUCs) were constructed for factors independently associated with bacterial yield. Analyses were performed with IBM SPSS Statistics 28 (IBM Corporation, New York, USA, 2021).

### Use of AI

During the preparation of this work the authors used OpenAI’s ChatGPT and Microsoft Copilot to improve language, grammar and clarity in the manuscript. After using these tools, the authors reviewed and edited the content as needed and take full responsibility for the content of the publication.

## Results

Of 471 enrolled patients, 174 patients with confirmed influenza were included (Fig. 1). Ninety patients (51.7%) were positive for influenza A, and eighty-four patients (48.0%) were positive for influenza B.

### Patient characteristics

Patient characteristics of 174 included patients who did and did not receive antibiotics at the ED, as well as did or did not have a bacterial coinfection are presented in Table 1. Forty-eight patients (27.6%) were admitted to hospital. Two additional patients were admitted within one week after inclusion but were not classified as admitted per protocol. They did not receive antibiotics and did not have bacterial coinfection. No patient was treated at intensive care unit and one patient died within 30 days. Chest radiology was performed in 63 of 174 patients (36.2%), of whom 24 (38.1%) had an infiltrate consistent with pneumonia.

Forty-one patients (23.6%) received antibiotic therapy, see Additional file 2: Table S1 for details. Six (14.6%) patients had their treatment discontinued within five days. Of 133 patients who did not receive antibiotics at the ED, 17 (12.8%) were started on antibiotic therapy within seven days.

In 98 (56.3%) patients, microbiological testing was performed, of whom 17 (17.3%) were identified as having a bacterial coinfection (Table 3). Detailed microbiological results are presented in Additional file 2: Table S2.

**Table 1:** Characteristics of patients with or without antibiotics and bacterial coinfection

	Antibiotics, *n* = 41	No antibiotics, *n* = 133	*p-value*	Bacterial coinfection, *n* = 17	No bacterial coinfection, *n* = 81	*p-value*
*Patient characteristics*						
Age	67 (18–93)	49 (18–88)	**0.02**	54 (21–93)	58 (18–92)	0.60
Female sex	23/41 (56.1)	61/133 (45.1)	0.22	8/17 (47.1)	35/81 (43.2)	0.77
Vaccinated for influenza	13/41 (31.7)	35/130 (26.9)	0.55	6/17 (35.3)	26/80 (32.5)	0.82
mCCI	1 (0–5)	1 (0–4)	0.10	1 (0–2)	1 (0–4)	**0.04**
COPD	3/41 (7.3)	10/133 (7.5)	1.00	0/17 (0.0)	5/81 (6.2)	0.58
Symptoms ≥ 10 days	1/41 (2.4)	5/133 (3.8)	1.00	0/17 (0.0)	4/81 (4.9)	1.00
*Parameters at inclusion*						
Respiratory rate^a^	22 (12–41)	20 (16–40)	**< 0.01**	20 (16–30)	20 (12–40)	0.66
Saturation^b^	94 (85–100)	97 (84–100)	**< 0.01**	96 (85–100)	96 (84–100)	0.51
Systolic blood pressure^c^	140 (80–212)	131 (90–190)	0.39	130 (80–212)	139 (99–207)	0.27
Diastolic blood pressure^d^	80 (50–114)	80 (50–117)	0.10	80 (60–100)	80 (50–117)	0.62
Heart rate	105 (67–157)	92 (62–146)	**< 0.01**	102 (68–157)	100 (63–150)	0.19
Acute confusion	0/41 (0.0)	0/132 (0.0)	n/a	0/17 (0.0)	0/81 (0.0)	n/a
Body temperature^e^	38.6 (36.6–40.6)	38.5 (35.8–40.5)	0.17	38.5 (36.6–40.4)	38.6 (35.8–40.6)	0.82
NEWS2 ≥ 7 points	14/41 (34.1)	5/133 (3.8)	**< 0.01**	4/17 (23.5)	11/81 (13.6)	0.29
CRB-65 ≥ 2 points	10/41 (24.4)	3/133 (2.3)	**< 0.01**	3/17 (17.6)	8/81 (9.9)	0.40
CRP (mg/L)	64 (3–239)	26 (1–204)	**< 0.01**	71 (22–210)	34 (4–239)	**< 0.01**
WBC (x10^9^/L)^f^	8.7 (4.2–29.0)	6.1 (1.0–14.5)	**< 0.01**	8.0 (5.0–29)	7.1 (2.8–23.1)	0.18
ANC (x10^9^/L)^g^	6.9 (3.2–25.0)	4.2 (1.2–11.3)	**< 0.01**	5.9 (3.3–25.0)	5.3 (1.2–20.9)	0.26
ALC (x10^9^/L)^h^	0.9 (0.3–3.0)	0.9 (0.1–2.5)	0.48	1.0 (0.4–3.0)	0.9 (0.1–2.5)	0.06
*Diagnostics*						
Influenza B	22/41 (53.7)	62/133 (46.6)	0.43	9/17 (52.9)	43/81 (53.1)	0.99
Bacterial coinfection	10/34 (29.4)	7/64 (10.9)	**0.02**	--	--	--
Pulmonary infiltrate	17/31 (54.8)	7/32 (21.9)	**< 0.01**	3/9 (33.3)	18/45 (40.0)	1.00
*Treatment and outcome*						
Antibiotics used	--	--	--	10/17 (58.5)	24/81 (29.6)	**0.02**
Antivirals used	6/41 (14.6)	22/132 (16.5)	0.76	2/17 (11.8)	20/80 (25.0)	0.34
Admission	22/41 (53.7)	26/133 (19.5)	**< 0.01**	7/17 (41.2)	37/81 (45.7)	0.73
Oxygen support	9/41 (22.0)	6/133 (4.5)	**< 0.01**	3/17 (17.6)	11/81 (13.6)	0.71
HFNO	1/41 (2.4)	0/133 (0.0)	0.24	0/17 (0.0)	1/81 (1.2)	1.00
CPAP/BiPAP	1/41 (2.4)	0/133 (0.0)	0.24	1/17 (5.9)	0/81 (0.0)	0.17
30-day mortality	1/41 (2.4)	0/133 (0.0)	0.24	0/17 (0.0)	1/81 (1.2)	1.00

Variables are shown as median (range) or proportions (%). mCCI: modified Charlson Comorbidity Index; COPD: chronic obstructive pulmonary disease; NEWS2: National Early Warning Score 2; CRB-65 = confusion, respiratory rate, blood pressure and age ≥ 65 years; CRP: C-reactive protein; WBC: white blood cell count; ANC: absolute neutrophile count; ALC: absolute lymphocyte count; HFNO: high-flow nasal oxygen; CPAP: continuous positive airway pressure; BiPAP: bilevel positive airway pressure

^a^Ten missing values

^b^Three missing values

^c^Nine missing values

^d^Twenty-four missing values

^e^Two missing values

^f^Three missing values

^g^Forty-six missing values

^h^Forty-eight missing values

### Multivariable logistic regression analysis

Clinical and biochemical factors significantly associated with antibiotic initiation or bacterial coinfection in univariate analysis were included in multivariable logistic regression models (Table 2). Individual vital signs were not included due to potential collinearity. Pulmonary infiltrates were not included in the model due to few events. In a sensitivity analysis including pulmonary infiltrates, NEWS2, CRB-65 score and CRP level were independently associated with antibiotic initiation, whereas pulmonary infiltrates were not (data not shown).

**Table 2:** Logistic regression analysis of factors associated with antibiotic initiation and bacterial coinfection

	Univariate		Multivariable	
	OR (95% CI)	*p*-value	aOR (95% CI)	*p*-value
*Antibiotic use*				
Age	1.02 (1.00–1.04)	**0.02**	0.99 (0.97–1.02)	0.48
mCCI	1.35 (0.98–1.85)	0.07	1.50 (0.91–2.48)	0.11
Female sex	1.56 (0.77–3.15)	0.22		
Vaccinated for influenza	1.26 (0.59–2.70)	0.55		
COPD	0.97 (0.25–3.71)	0.97		
Symptoms ≥ 10 days	1.56 (0.18–13.77)	0.69		
NEWS2 ≥7 points	13.27 (4.41–39.97)	**< 0.01**	8.99 (2.38–33.96)	**< 0.01**
CRB-65 ≥ 2 points	13.99 (3.63–53.83)	**< 0.01**	13.22 (2.70–64.73)	**< 0.01**
CRP (+ 25 mg/L)	1.67 (1.35–2.07)	**< 0.01**	1.57 (1.21–2.02)	**< 0.01**
WBC (x10^9^/L)	1.33 (1.17–1.52)	**< 0.01**	1.25 (1.07–1.46)	**< 0.01**
*Bacterial coinfection*				
Age	0.99 (0.97–1.02)	0.54	1.00 (0.97–1.03)	0.90
mCCI	0.51 (0.26–1.00)	**0.05**	0.41 (0.18–0.94)	**0.04**
Female sex	1.17 (0.41–3.34)	0.77		
Vaccinated for influenza	1.13 (0.38–3.40)	0.82		
COPD	0.00 (0.00–0.00)	1.00		
Symptoms ≥ 10 days	0.00 (0.00–0.00)	1.00		
NEWS2 ≥7 points	1.96 (0.54–7.10)	0.31		
CRB-65 ≥ 2 points	1.96 (0.46–8.29)	0.36		
CRP (+ 25 mg/L)	1.37 (1.11–1.69)	**< 0.01**	1.47 (1.16 − 1.86)	**< 0.01**
WBC (x10^9^/L)	1.09 (0.97–1.22)	0.14		

mCCI: modified Charlson Comorbidity Index; COPD: chronic obstructive pulmonary disease; NEWS2: National Early Warning Score 2; CRB-65 = confusion, respiratory rate, blood pressure and age ≥ 65 years; CRP: C-reactive protein; WBC: white blood cell; OR, odds ratio; CI, confidence interval; aOR, adjusted odds ratio

### Diagnostic accuracy

**Fig. 2:**
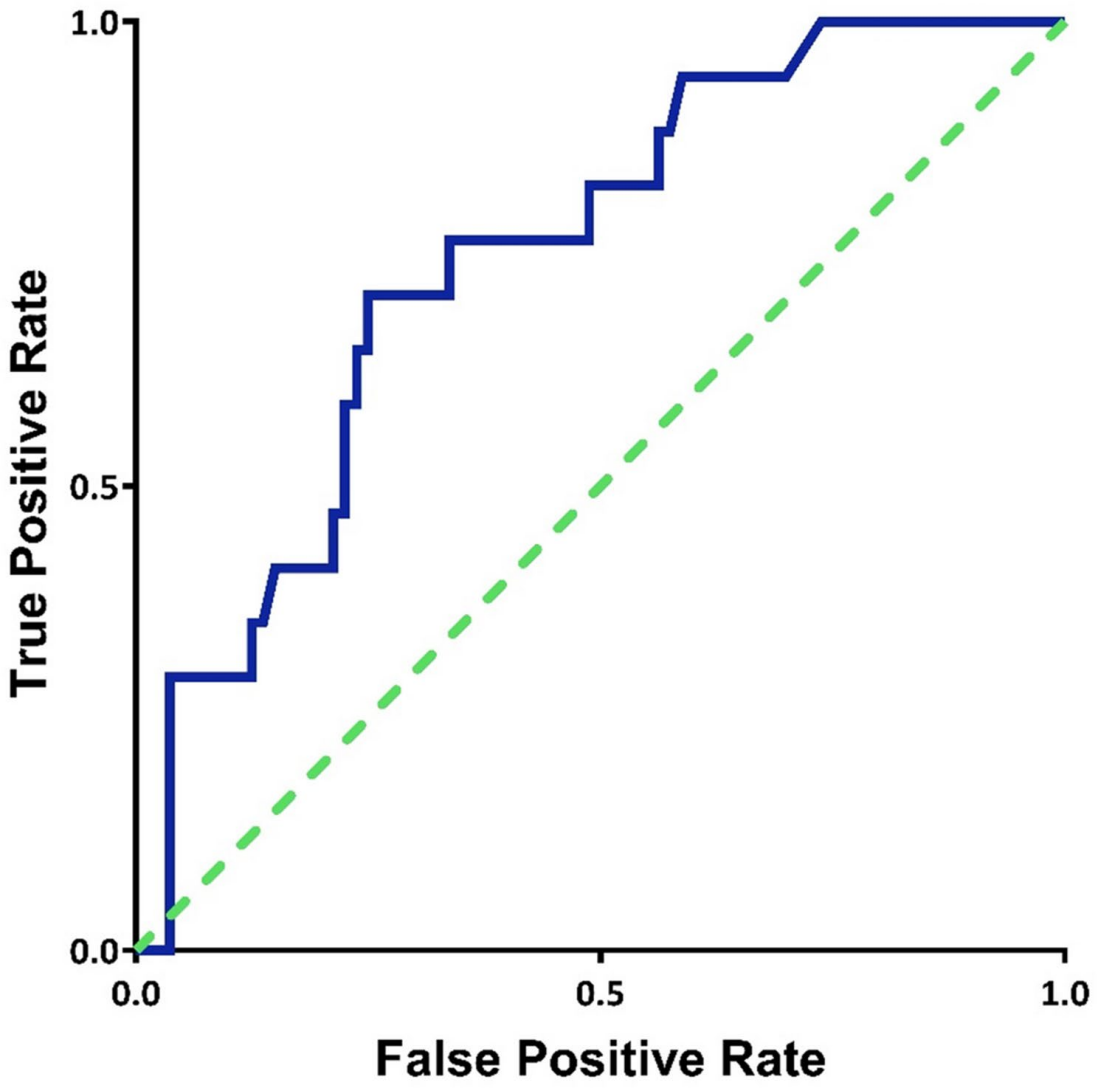
The ROC curve plot for CRP as a test of identifying bacterial coinfection, AUC 0.75

Given that CRP level was independently associated with bacterial coinfection, its diagnostic performance was assessed using ROC curve analysis (Fig. 2). CRP discriminated between pure viral infection and bacterial coinfection with an AUC of 0.75 (95% CI: 0.64–0.87). Sensitivity, specificity, positive predictive value (PPV) and negative predictive value (NPV) for different CRP cutoffs are presented in Table 3. A threshold aiming for at least 95% diagnostic certainty resulted in a lower CRP cutoff of 20 mg/L, yielding 100% sensitivity (95% CI: 80.5–100%), whereas a higher cutoff of 150 mg/L yielded 95.1% specificity (95% CI: 87.8–98.6%).

**Table 3:** Diagnostic accuracy of CRP for identifying bacterial coinfection

CRP cutoff	Sensitivity (%)	Specificity (%)	PPV (%)	NPV (%)
CRP ≥ 20	100	25.9	22.1	100
CRP ≥ 30	94.1	42.0	25.4	97.1
CRP ≥ 40	76.7	58.0	27.7	92.2
CRP ≥ 50	70.6	71.6	34.3	92.1
CRP ≥ 60	70.6	75.3	37.5	92.4
CRP ≥ 70	52.9	77.8	33.3	88.7
CRP ≥ 80	47.1	77.8	30.8	87.5
CRP ≥ 90	41.2	83.9	35.0	87.2
CRP ≥ 100	35.3	87.7	37.5	86.6
CRP ≥ 110	29.4	87.7	33.3	85.5
CRP ≥ 120	29.4	92.6	45.5	86.2
CRP ≥ 130	29.4	92.6	45.5	86.2
CRP ≥ 140	29.4	93.8	50.0	86.4
CRP ≥ 150	29.4	95.1	55.6	86.5

CRP: C-reactive protein; PPV: positive predictive value; NPV: negative predictive value

## Discussion

In this study of patients with laboratory-confirmed influenza presenting to the ED, we found that high clinical scores (NEWS2 and CRB-65) and elevated biomarkers (CRP and WBC count) were independently associated with antibiotic initiation, while only CRP level was independently associated with bacterial coinfection. Our findings suggest that clinicians initiate antibiotic therapy in patients with pure influenza who are severely ill, either because they believe a bacterial infection is likely or at least cannot rule it out when microbiological test results are unavailable at the ED. CRP levels may help discriminate between pure viral infection and bacterial coinfection, but the use of a specific cutoff for guiding antibiotic management needs to be tested prospectively and in combination with other clinical and biochemical factors.

### Antibiotic initiation

In our study, antibiotics were initiated in 23.6% of all cases. In comparison, Taymaz et al. reported a similar initiation rate of 21.7% among influenza-positive patients, in a retrospective study of patients with influenza-like illness in the ED [11]. However, Roth et al. found a higher antibiotic initiation rate (38.2%) in influenza-positive patients, which may be due to an older population compared to our study [12]. In a Swedish study by Bergbrant et al. on hospitalized influenza patients specifically, they reported a 56.5% antibiotic prescription rate, compared with 51.2% (22/48; Table 1) in our admitted patients [23]. The difference may reflect lower overall disease severity in our population, including no ICU admissions, compared to 6.8% in the Bergbrant cohort.

To our knowledge, this is the first study to examine predictors of antibiotic initiation exclusively in patients with laboratory-confirmed influenza, describing independent associations with both clinical scores and biomarkers. In a retrospective study by Debes et al., CRP levels and vital signs were independently associated with antibiotic use, consistent with our findings [14]. They also found associations with clinical scores NEWS and CRB-65, however, these were not included in the multivariable model. Taymaz et al. reported independent associations between antibiotics and elevated CRP levels and WBC counts, but did not investigate vital signs or clinical scores [11]. In contrast with our findings, these studies also observed independent associations with age and comorbidities [11, 14], although, influenza negative patients were included as well and may imply that antibiotics are more likely to be initiated in elderly and comorbid patients when the influenza test is negative.

Previous studies have reported independent associations with pulmonary infiltrates on CXR [11, 12, 14], suggesting that pulmonary imaging may play an important role in antibiotic therapy decisions. In our study, pulmonary infiltrates were associated with antibiotic initiation in univariate analysis but were not included in the multivariable model due to the limited number of events. However, when pulmonary infiltrates were incorporated into a sensitivity analysis of the multivariable model, no independent association was observed. This may indicate that clinicians do not consider pulmonary infiltrates when deciding on antibiotic initiation in patients with confirmed influenza specifically, although it may also reflect the small number of events. Therefore, this result should be interpreted with caution.

### Bacterial coinfection

Reported rates of bacterial coinfection in influenza vary widely, from 1.3% to 75%, whereas pooled prevalence estimates range between 11.2% and 20.3% [4, 5]. The variability likely reflects differences in study design, reference standards, and patient selection criteria. In comparison, the rate of bacterial coinfection was 17.3% in our cohort. Bergbrant et al., using a similar reference standard, reported a slightly lower rate of 11.7% in hospitalized patients with influenza [23]. Other retrospective Swedish studies have reported rates ranging from 9.4% in patients with seasonal influenza A, to 27% in hospitalized patients with influenza and radiologically confirmed pneumonia [24, 25].

The search for accurate diagnostic tools to distinguish bacterial from viral RTIs has been ongoing for years [8]. A major challenge is the lack of standardized criteria for identification of bacterial etiology in RTIs. Findings from studies with similar reference standards as in ours are consistent with our results. For instance, Ahn et al. conducted a small retrospective study of hospitalized patients with 2009 H1N1 influenza pneumonia and reported a univariate association between CRP levels and bacterial coinfection, while vital signs showed no association [16]. Haran et al., who used pulmonary infiltrates and bacterial diagnosis at discharge as reference standard, likewise found an association with CRP and not with vital signs [7]. Moreover, Bergbrant et al. demonstrated that high NEWS scores were more common in pure viral infections, further underscoring the weak correlation between abnormal vital signs and bacterial infection [26].

### Diagnostic accuracy

As CRP emerged as the strongest independent predictor of bacterial coinfection in our study, we further evaluated its diagnostic accuracy. Few studies have assessed the accuracy of CRP specifically among patients with laboratory-confirmed influenza. In our cohort, CRP demonstrated an AUC of 0.75 for detecting bacterial coinfection, indicating moderate diagnostic performance consistent with prior findings. In comparison, Ahn et al. reported a similar AUC of 0.70, whereas Li et al. found an AUC of 0.76 in a pediatric H1N1 population [15, 16]. Haran et al. reported a higher AUC of 0.98; however, they used a different reference standard and included cases of primary bacterial infection as well [7].

### Clinical relevance

Our findings indicate that clinicians may interpret high clinical scores as indicators of bacterial coinfection, despite their limited predictive value in this population. However, based on our results, clinicians could likely withhold antibiotic therapy if CRP levels are low, even when patients with influenza have high clinical scores. A CRP cutoff of 20 mg/L may serve as a rule-out value for bacterial coinfection, while a cutoff of 150 mg/L could function as a rule-in value, although the clinical usefulness of these cutoffs may be limited. Our findings could improve antibiotic stewardship strategies by underscoring the limited predictive value of clinical scores and optimal use of biomarkers.

### Limitations

Our study has several limitations. First, this is a sub-analysis of a prospectively enrolled cohort which was originally designed with a different primary aim [17], and consequently many outcomes and exposures were extracted retrospectively. While most variables were standardized measurements obtained from all participants, antibiotic prescription, microbiological testing (including complete set of cultures and molecular bacterial diagnostics) as well as chest radiography were not performed in all patients as per protocol. Therefore, we recommend prospective studies including all variables to confirm our results. Second, since coinfections were identified mainly based on positive results from nasopharyngeal cultures, bacterial colonization may be a risk for overestimation of coinfections. However, the expected rate of colonization in our study population was low, based on a previous report of 3% pneumococcal carriage on adult healthy controls in our region [27]. Additionally, we found a strong association between elevated CRP levels and bacterial yield, suggesting these findings represent more than mere colonization. Third, the overall mortality in our study is lower than in other comparable studies [14–16, 23]. This is likely due to the prospective nature of the cohort and the challenges of including critically ill patients when they are not the target population. As a result, our findings cannot be generalized to critically ill patients at the ER. Finally, although this is one of the largest clinical studies on antibiotic use in influenza patients, most patients were non-severely ill and were recruited from only two hospitals, which limits the generalizability of our findings.

## Conclusions

The clinical scores NEWS2 and CRB-65, as well as the biomarkers CRP and WBC, were independently associated with antibiotic initiation in patients with confirmed influenza presenting to the ED. CRP was the only factor independently associated with bacterial coinfection. These findings may help improve ED-based decision tools for antibiotic stewardship in influenza. Larger prospective studies incorporating comprehensive microbiological and radiological assessment, as well as interventional trials with antibiotic strategies as per protocol, are needed to validate the clinical applicability of our findings.

## Supplementary Information

Below is the link to the electronic supplementary material.

Supplementary Material 1

Supplementary Material 2

## Data Availability

The datasets used and/or analysed during the current study are available from the corresponding author on reasonable request.

## Abbreviations

AUC: Area under the curve
CI: Confidence interval
CRB-65: Confusion, respiratory rate ≥ 30, blood pressure systolic < 90 or diastolic ≤ 60 and age ≥ 65 years
CRP: C-reactive protein
CXR: Chest X-ray
ED: Emergency department
mCCI: Modified Charlson Comorbidity Index
NEWS2: National Early Warning Score 2
NPV: Negative predictive value
PPV: Positive predictive value
ROC: Receiver operating characteristic
RT-PCR: Reverse transcription polymerase chain reaction
RTI: Respiratory tract infection
WBC: White blood cell
