# Supplementary material for: Factors associated with antibiotic initiation and bacterial coinfection in adults with confirmed influenza
Source: BMC Infect Dis. 2026 Mar 25;26:742. doi: 10.1186/s12879-026-13166-0 (PMC13063665; doi:10.1186/s12879-026-13166-0)
Supplement: Supplementary file 1 — Supplementary Material 1 [file 12879_2026_13166_MOESM1_ESM.pdf]

Personal identity number

Name

Space for label

Label from sampling requisitions

QUESTIONNAIRE

Myocardial Involvement During Influenza

General Information

Date of inclusion:____________________

Height: _______cm

Weight: _________kg

ECG performed: Yes/No

Blood sample –ROS referral for *HJINF* study sent: Yes/No

NPH sample sent: Yes/No

Signs of infection

- Number of days with current infectious symptoms:_________________________
- Highest measured temperature during the current illness: __________________C

- Vaccinated against influenza this season: Yes/No
- Date of vaccination (if applicable): ____

Background

- Smoker / former smoker / non-smoker

Previously diagnosed with:

• Hypertension:		Yes/No
• Hyperlipidemia:		Yes/No
• Diabetes Mellitus:	Oral medication	Yes/No
	Insulin	Yes/No
• Myocardial infarction:		Yes/No
• Heart failure:		Yes/No
• Myocarditis		Yes/No
• Stroke:		Yes/No
• Claudication:		Yes/No
• COPD:		Yes/No
• Asthma:		Yes/No

Signature:________________________
