# Supplementary material for: Factors associated with antibiotic initiation and bacterial coinfection in adults with confirmed influenza
Source: BMC Infect Dis. 2026 Mar 25;26:742. doi: 10.1186/s12879-026-13166-0 (PMC13063665; doi:10.1186/s12879-026-13166-0)
Supplement: Supplementary file 2 — Supplementary Material 2 [file 12879_2026_13166_MOESM2_ESM.docx]

## Additional file 2

**Table S1**

Antibiotics used at the emergency department

|  | n |  |  |  |
| --- | --- | --- | --- | --- |
| Amoxicillin | 6 |  |  |  |
| Bencylpenicillin | 11 |  |  |  |
| Cefotaxime | 7 |  |  |  |
| Doxycycline | 7 |  |  |  |
| Fenoxymetylpenicillin | 6 |  |  |  |
| Levofloxacin | 1 |  |  |  |
| Piperacillin and tazobactam | 2 |  |  |  |

**Table S2**

Microbiological testing and relevant findings

| Bacteria by standard culture | Blood (n=82) | NPH (n=84) | Sputum (n=8) |
| --- | --- | --- | --- |
| *Streptococcus pneumoniae* | 0 | 8 | 0 |
| *Haemophilus influenzae* | 0 | 8 | 0 |
| *Moraxella catarrhalis* | 0 | - | 1 |
| *Fusobacterium necrophorum* | 1 | - | 0 |
|  |  |  |  |
| Bacteria by PCR | Throat (n=2) | Sputum (n=0) |  |
| *Mycoplasma pneumoniae* | 0 | 0 |  |
| *Chlamydia pneumoniae* | 0 | 0 |  |
| *Chlamydia psittaci* | 0 | 0 |  |
|  |  |  |  |
| Bacteria by antigen test | Urine |  |  |
| *Streptococcus pneumoniae* (n=8) | 0 |  |  |
| *Legionella pneumophila* (n=4) | 0 |  |  |
|  |  |  |  |
